# Theoretical basis and occurrence of internet fraud victimisation: Based on two systems in decision-making and reasoning

**DOI:** 10.3389/fpsyg.2023.1087463

**Published:** 2023-02-06

**Authors:** Yuxi Shang, Kaijie Wang, Yuye Tian, Yingyu Zhou, Beibei Ma, Sanyang Liu

**Affiliations:** ^1^School of Law, Shandong Normal University, Jinan, China; ^2^MBA Education Center, Shandong University of Technology, Zibo, China; ^3^Junde Experimental School, Jinan, China; ^4^School of Law, Jiangsu Normal University, Xuzhou, China

**Keywords:** internet fraud victims, the heuristic-systematic model, influencing factors, measure, defence strategies

## Abstract

The influencing factors of internet fraud, including demographics, psychology, experience and knowledge of susceptibility, have been widely studied. Research on the psychological mechanism of the victimisation process of internet fraud is relatively scarce but suggests a new research perspective. To summarise and unify the research in this field, this study systematically searched and analysed articles on the psychological decision-making mechanism of online fraud victims. We found that (a) previous researchers consistently believed that the heuristic processing mode was correlated with susceptibility to online fraud and that the systematic processing mode was helpful to detect and identify fraud. From the overall review results, we do not reject this conclusion, but the verification and intrinsic explanation of this relationship need to be further strengthened. (b) Under the heuristic-systematic model (HSM), with the exception of the trait of suspicion, there is no consensus on whether psychological factors (e.g., personality) influence the likelihood of online fraud through the mediating effect of the selection of the two systems. Objective knowledge and experience in specific fields have been found to be able to achieve this path. Information on the influential variables of equipment and habits is emerging, but how they affect network victimisation through the heuristic processing system needs to be further clarified. (c) The measurement of variables is conducted through simulation experiments. There may be a gap between the likelihood of internet fraud victimisation in the simulation experiment and in the real world. (d) The defence strategies under the HSM are intentional explorations, such as content-based cue recognition technology and simulated scene training.

## Introduction

Internet fraud is defined as the act of obtaining money through deception using network communication technology or the act of providing fraudulent invitations to potential victims or conducting fraudulent transactions using the internet (Tade and Aliyu, 2011; Whitty, 2015, 2019; Gao, 2021). Internet fraud is also called phishing and is typically performed by sending victims an email that is ostensibly from a legitimate organisation or individual (Frauenstein and Flowerday, 2020). With the communication technologies currently available, especially mobile devices, internet fraud occurs not only through email but also through text messages, social networking sites (SNSs), and telephones (Vishwanath, 2015; Aleroud and Zhou, 2017; Frauenstein and Flowerday, 2020).

Internet fraud, including phishing, is the fifth most common cause of security incidents and has the highest success rate of any threat vector (Verizon, 2019). Facebook and Google were defrauded of more than $100,000 through a phishing scheme that impersonated a large Asian-based manufacturer in 2017 (United States Department of Justice, 2017). A meta-analysis showed that internet fraud in the United States in 2018 caused approximately 2.7 billion dollars in economic losses. Internet fraud is also the fastest growing crime in the United Kingdom, with approximately 3.25 million people becoming victims each year (Norris et al., 2019). At the beginning of the COVID-19 pandemic in 2020, online fraudsters began to take advantage of people’s panic and uncertainty to conduct phishing attacks (Muncaster, 2020). Internet fraud has become an important social governance problem (Burnes et al., 2017) and has attracted increasing attention from scholars (Vishwanath et al., 2011; Modic and Lea, 2013; Harrison et al., 2016a; Modic et al., 2018).

Routine activity theory notes that victimisation is caused by motivated criminals, appropriate targets and a lack of effective guardianship (Cohen and Felson, 2010). Motivated criminals use cunning as a means of defrauding victims. Cialdini (2018) has summarised six key principles of persuasion often used by fraudsters: reciprocity, social proof or conformity, commitment or consistency, authority, liking, and scarcity. For example, when confronted with scarcity information, the receiver responds to the information to avoid the loss of opportunities (Bullée et al., 2015). In terms of effective guardianship, all countries attach great importance to combating and preventing internet fraud. Common means include strict legal action against criminals, educating and reminding potential victims, and interception by technical methods (Chen and Yang, 2022). Questioning why many people every day suffer from internet fraud attacks requires a shift of vision to the appropriate target or victim. There are three main directions for research on potential victims of internet fraud.

The first research direction is demographics, which refers to the relationship between the age, income, education, gender, and race of victims of internet fraud (Cohen et al., 1981; Holtfreter et al., 2006; Salthouse, 2012; Burnes et al., 2017; Gavett et al., 2017). Carcach et al. (2001) found that men are more likely than women to be victims of personal crimes such as internet fraud. Age has been the focus of many scholars’ attention and research, and the growing ageing phenomenon and the spread of anecdotal evidence, such as news reports, have formed the concept that older adults are more vulnerable to fraud. Many scholars have analysed different factors of internet fraud victims and found that compared with other types of crimes, older adults are more likely to become victims of consumer fraud (Carcach et al., 2001). Burnes et al. (2019) agreed that “the elderly are more easily cheated, which is related to their slow cognitive processing and high experiences of loneliness.” In addition, James et al. (2014) found that vulnerability to fraud is related to victims’ income and education level. Some studies by the Federal Trade Commission (FTC) show that Aboriginal Americans, African Americans, and Hispanic Americans are more likely than non-Hispanic white Americans to be victims of fraud (Anderson, 2004; Anderson, 2013).

Second, regarding the direction of psychological characteristics, researchers have mainly studied the influencing factors of susceptibility to online fraud. These include risk perception (Moody et al., 2017), trust (Wright and Marett, 2010), suspicion (Harrison et al., 2016a), personality (Ashton and Lee, 2009), and self-control (Modic and Lea, 2012). Holtfreter et al. (2008) proposed that groups with low self-control are more likely to be cheated. This is mainly because people with low self-control attempt to meet their needs immediately. They may follow the instructions of a fraudster to obtain a promise. In research on the relationship between personality and vulnerability to online fraud, researchers found that not all personality traits predict vulnerability to fraud. Alseadoon et al. (2012) simulated fraud against 200 college students and found that openness and extraversion could improve the possibility of replying to emails, although no other personality traits were found to have a predictive effect. In the study of personality differences and susceptibility to online fraud, scholars have also examined the relationship between victims’ online experience, security knowledge and susceptibility to online fraud (Larcom and Elbirt, 2006; Wright and Marett, 2010).

Third, with regard to the direction of the psychological mechanism, according to interpersonal deception theory, fraud is essentially antagonistic to social interaction, which requires cognitive resources (Buller and Burgoon, 1996). Deception works because the deceiver takes advantage of the target’s weakness in information processing and takes measures to thwart the target’s cognitive efforts in interaction (Johnson et al., 2001). In other words, the target is victimised because of a weakness in information processing, failure in the cognitive detection of fraudulent information, or both. Previous studies have confirmed that users’ cognitive processing is a key cause of individual online fraud victimisation (Vishwanath et al., 2011). Related theories are the heuristic-systematic model (HSM), the elaboration likelihood model (ELM), and the theory of deception:

The HSM is a model of information processing that includes two information processing modes: the heuristic system based on intuition and the analytic system based on rationality (Chaiken, 1980; Sloman, 1996; Evans, 2003). The Heuristic system relies more on intuition; parallel processing is fast and does not occupy or occupies little psychological resources. The Analytic System relies more on rationality, serial processing is slow, and occupies more psychological resources. The study also found that heuristic processing leads to lower risk assessment (Tversky and Kahneman, 1974; Trumbo, 2002), which makes it difficult for people to identify the traps in the fraudulent information and ultimately leads people to suffer fraud. Phishing attacks usually increase their success rate by misleading the target victim to make a quick but incorrect evaluation of information effectiveness (Luo et al., 2013).

The ELM is also a dual process model; it distinguishes between two ways in which individuals process information. The central processing route involves careful consideration of presented information using comparisons and prior experience, but the peripheral processing route does not consider all elements of the message (Petty and Cacioppo, 1986). Although the HSM is theoretically similar to the ELM, the HSM emphasizes that two distinct modes of thinking about information can occur, and the ELM suggests that information processing occurs on a continuum instead (Frauenstein and Flowerday, 2020). According to Petty and Cacioppo, information processing activities include two subprocesses: attention and elaboration. Attention is the first stage in information processing and indicates the amount of mental focus given to specific elements of an event or object (Eveland et al., 2003; Vishwanath et al., 2011). Elaboration is the process through which individuals make conscious connections between the cues they observe and their prior knowledge (Perse, 1990; Vishwanath et al., 2011). Jakobsson (2007) found that the target was victimised, probably because certain cues in a phishing e-mail address (e.g., e-mail address) were not noticed. Users who can identify fraud are able to pay attention to irrational clues (ELM’s attention process) and use previous experience and knowledge for evaluation (ELM’s elaboration process).

The theory of deception is also known as the detecting deception model. It refers to individuals identifying fraud by noticing and interpreting inconsistencies between anomalies and their past experience; thus, clue processing is further elaborated (Johnson et al., 1992, 2001). According to the detection deception model, the process of identifying fraud can be divided into four stages: a. Activation, detecting anomalies of fraud information. b. Hypothesis generation, interpreting abnormal clues and generating suspicion. c. Hypothesis evaluation, comparing the hypotheses developed in the previous stage with certain criteria. d. Global assessment, combination and overall evaluation of known clues. These four stages of cognitive effort are similar to the process of elaboration (Eveland et al., 2003; Vishwanath et al., 2011). In 2004, Grazioli tested the authenticity of the trading site on Eighty MBA students and found that competence in evaluating the hypothesis of deception (stage c) was a strong differentiator between successful and unsuccessful detection. Although a large number of previous studies have been conducted on the relationship between demographics, psychological traits and online fraud, there is no consensus on the research conclusions. For example, regarding demographic factors, Button et al. (2009) proposed that no demographic characteristic is necessarily more or less susceptible to internet fraud. Shang et al. (2022) found that elderly people were not a susceptible population, and the influencing factors measured in the past were untenable based on a systematic review of the literature. Regarding psychological factors, there are mutually exclusive research results in relation to trust and other factors (McKnight et al., 2004; Judges et al., 2017). Research on online fraud should examine the decision process of victims in the face of fraudulent information (Norris et al., 2019).

A summary of the decision process of network fraud victims shows that although the ELM distinguishes the central processing route (system 1) and the peripheral processing route (system 2) in theory, there is no measurement or classification of these two systems in practice. Researchers mainly focus on the relationship between two subsystems of ELM (attention and elaboration) and online fraud (Vishwanath et al., 2011; Harrison et al., 2016b). Attention and elaboration are often regarded as indicators of the systematic processing of HSM (Frauenstein and Flowerday, 2020; Gao, 2021). Therefore, our research vision should be on ELM. This study searched and analysed the literature on the decision process of online fraud victims using the heuristic systematic model to obtain and discuss previous research conclusions on the victimisation process and promote further exploration in this field.

## Materials and method

### Systematic review

This manuscript is a systematic review, and the scope of the review is the literature on the information processing model of internet fraud victims, particularly the heuristic-systematic model. A systematic review is different from a meta-analysis; while the current research literature on fraud victimisation mentions the heuristic or systematic processing mode (in addition to the analytic processing mode), there is little research on the correlation between the two processing modes and fraud susceptibility. Specifically, the previous literature focuses either on the relationship between the information processing mode and trust, doubt and susceptibility or on the relationship between the subprocesses of the cognitive processing mode (attention and elaboration) and the above dependent variables. In other words, published studies on the processing modes of network victimisation differ in terms of the independent variables, dependent variables, intervention methods and research design, which makes it difficult to meet the prerequisite conditions for meta-analysis (Cheung and Vijayakumar, 2016).

### Search strategy

This study was conducted using guidelines and checklists outlined by the Preferred Reporting Project for Systematic Review and Meta-Analysis (PRISMA) group (Moher et al., 2009). This search was based on relevant full-text articles selected from multiple database searches of all published documents from the establishment of each database to May 2022 (search process updated on 16 October 2022). The following English databases were used: Web of Science Core Collection, Elsevier, SciELO Citation Index2, ProQuest, and PsycArticles. The English search strategy was as follows: (phishing email OR phishing OR phished OR online OR internet OR cyber OR network OR telemarketing) AND (fraud OR cheat OR swindle OR scam OR deception OR susceptibility to scam OR susceptibility to deception OR susceptibility to persuasion OR susceptibility to fraud OR phishing vulnerability OR phishing susceptibility OR fraud victims OR phishing victims) AND (cognition OR cognitive processing OR information processing OR heuristic model OR systematic model OR HSM OR system processing OR elaboration likelihood model OR ELM OR elaboration OR processing clues OR attention OR suspicion). To more clearly express our search strategies, we have set up Table 1.

**Table 1:** Search Strategy.

Items	Content
Search specification	Guidelines and checklists outlined by the Preferred Reporting Project for Systematic Review and Meta-Analysis (PRISMA) group (Moher et al., 2009).
Databases	Web of Science Core Collection, Elsevier, SciELO Citation Index2, ProQuest and PsycArticles.
Search time	All relevant articles published from the inception of each database until May 2022 (search process updated on 16 October 2022).
Search keywords	Phishing email, phishing, etc.; online, internet, etc.; susceptibility to fraud, phishing victims, etc.; information processing, HSM, etc.
Search formula	(Phishing email OR phishing OR phished OR online OR internet OR cyber OR network OR telemarketing) AND (fraud OR cheat OR swindle OR scam OR deception OR susceptibility to scam OR susceptibility to deception OR susceptibility to persuasion OR susceptibility to fraud OR phishing vulnerability OR phishing susceptibility OR fraud victims OR phishing victims) AND (cognition OR cognitive processing OR information processing OR heuristic model OR systematic model OR HSM OR system processing OR elaboration likelihood model OR ELM OR elaboration OR processing clues OR attention OR suspicion)
Search result	6,835 relevant articles were obtained by eliminating duplicate articles

### Inclusion criteria and exclusion criteria

The topic of this paper is the information processing mode of internet fraud victims. In terms of article types, experimental or measurement studies were preferred, and explanatory phenomenological analysis and anecdotal comments on cases and scams were excluded. For systematic review studies, only the full text of literature that discussed the information processing methods of internet fraud was chosen.

In addition to the investigation of research topics, the following types of studies were excluded: (1) not in a peer-reviewed journal; (2) written in any language other than English; (3) full text could not be accessed through the university library or obtained directly from the corresponding author; (4) published in abstract form (failure to provide enough information to analyse the impact of information processing modes on victims); and (5) used qualitative research methods.

### Article screening

**Figure 1:**
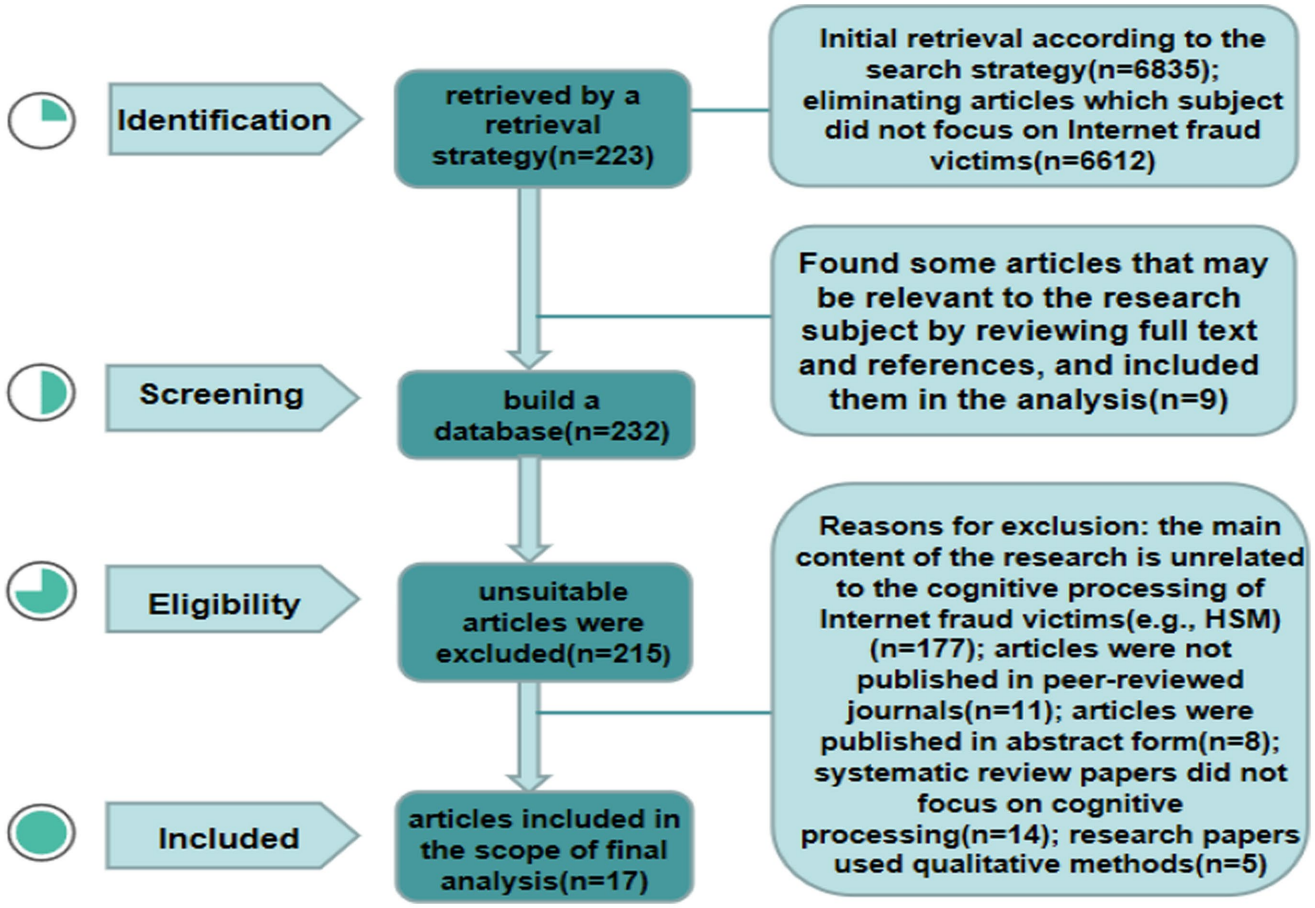
Identification flowchart of the search for literature on cognitive information processing of online fraud victims (model drawings comes from the author’s construction).

Under the guidance of the search strategy, 6,835 relevant articles were obtained by eliminating duplicate articles. The objects of this study were victims of online fraud. Article titles and abstracts were searched, and 6,612 articles were found that did not focus on the subject of online fraud victims. For the remaining 223 articles, the full text and references of these articles were checked. We found that 9 articles were not included in these 233 articles but may be possibly related to network fraud victims. We included these 9 articles and 233 articles previously screened into the analysis. Then, a database of 232 articles was built. According to the inclusion and exclusion criteria, especially the key words of cognitive information processing of network fraud victims, 17 articles were finally included in the analysis. (1) Although most of the literature mentioned the cognitive information processing process of network fraud victims (e.g., the HSM), discussion of the information processing mode was minimal and not the key object of the study in 177 articles, which were excluded. (2) Eleven articles were not published in peer-reviewed journals. (3) Eight articles were published in abstract form. (4) Fourteen systematic review papers did not focus on cognitive processing. (5) Five research papers used qualitative methods. See Figure 1 for the selection process.

### Quality assurance

The entire process of searching and screening was completed by two graduate students independently. To ensure the objectivity and accuracy of the screening, the research team first fully discussed the inclusion and exclusion criteria and unified opinions on the preset divergence. After the screening was finished, the screening tools were used to compare the results and conduct a collective study on the literature with different opinions. The selection and reporting of risks was controlled, to a certain extent, through the above process. Finally, the publishing risk of the final included articles was evaluated. Among the 17 articles included in the analysis, 94% of the published journals ranked in the top 50% of Journal Citation Reports.

## Results

The Supplementary Table shows the main characteristics of research on the psychological mechanism of online fraud victims using the HSM framework (N = 17). The following information was selected: source, country, method, sample size, sample description, and main findings. Researchers conducted the studies in the United States (*N* = 14; Grazioli and Wang, 2001; Johnson et al., 2001; Wright and Marett, 2010; Vishwanath et al., 2011, 2016; Wang et al., 2012; Luo et al., 2013; Petty and Briñol, 2014; Canfield et al., 2016; Vishwanath, 2016; Harrison et al., 2016a,b; Huang et al., 2022; Valecha et al., 2022), South Africa (N = 1; Frauenstein and Flowerday, 2020), China (*N* = 1; Chen and Yang, 2022), and the UK (*N* = 1; Jones et al., 2015).

Based on the similarity between the HSM and the ELM as well as the inclusion of the theory of deception, a systematic review of the cognitive processing mechanism of online fraud victims was conducted. We report the relationship between the HSM and susceptibility to online fraud. On this basis, the influencing factors of the decision mode selection of victims are discussed, and the network fraud defence countermeasures proposed by researchers under the HSM are highlighted.

### The selection of the heuristic-analytic processing mode and victims of internet fraud

Heuristic processing uses simple factors or messages (i.e., heuristic cues) to conduct rapid effectiveness evaluation, while systematic processing conducts a highly elaborative validity evaluation of the received information by carefully studying the content of the information and comparing the information with previous experience. This tendency to process information in different ways may influence users’ attitudes, judgements and behaviours towards specific information (Petty and Cacioppo, 1986). Studies have shown that individuals prefer heuristic processing rather than effort for information evaluation based on consideration of the cognitive resource economy (Sundar et al., 2007; Sundar, 2008). However, studies also show that heuristic processing leads to lower risk assessment, which makes it difficult for individuals to identify traps in fraudulent information and thus exposes people to fraud (Wang et al., 2012; Jones et al., 2015; Vishwanath et al., 2016).

The HSM argues that when people make a validity evaluation, their confidence in their evaluation must meet or exceed the adequacy threshold (the extent people wish to reach when making decisions) to feel comfortable with their own judgements (Eagly and Chaiken, 1993). When heuristic processing alone cannot guide message receivers to reach the sufficiency threshold, receivers are likely to invoke systematic processing (Luo et al., 2013). Vishwanath et al. (2016) found that systematic processing significantly reduces the chances of fraud victimisation; in contrast, heuristic processing significantly increases the chances of fraud victimisation, doubling the likelihood that people will be victims of email and Facebook phishing attacks. In addition, according to the weakening principle of the HSM, a high level of systematic processing can weaken the impact of heuristic processing and may even produce conclusions that limit or overturn heuristic processing (Watts and Zhang, 2008). When individuals activate system processing to detect and process fraud information, it is easier for them to identify online fraud (Grazioli and Wang, 2001; Grazioli, 2004).

The studies included in our analysis consistently indicate that victimisation through online fraud is related to the heuristic decision-making model. Phishing attackers know the weaknesses of human information processing and aim to improve the success rate of fraud by arousing victims’ heuristic thinking and reducing systematic thinking (Johnson et al., 2001; Luo et al., 2013; Canfield et al., 2016; Vishwanath et al., 2016; Chen and Yang, 2022). In terms of specific demonstrations, only 3 studies provided data analysis (other documents studied either the relationship between subsystems of the HSM and victimisation or the influencing factors of the HSM), which mainly demonstrated the information processing models (the heuristic processing mode vs. the systematic processing mode) and whether subjects were susceptible to online fraud (Table 2).

**Table 2:** Path analysis of heuristic-systematic processing and data results.

Authors	Path	*β*	*p*
Vishwanath et al. (2016)	Heuristic processing → Suspicion of phishing	Study 1: −0.04, Study 2: −0.17	=0.66 < 0.001
	Systematic processing → Suspicion of phishing	Study 1: 0.32 Study 2: 0.24	<0.05 < 0.001
Harrison et al. (2016a)	Heuristic processing → Trust in phishing	0.13	<0.10
	Systematic processing → Trust in phishing	−0.27	<0.05
Frauenstein and Flowerday (2020)	Heuristic processing → Phishing susceptibility	0.287	<0.001
	Systematic processing → Phishing susceptibility	−0.005	>0.05

Social psychology research on phishing suggests that an ineffective cognitive process is a major cause of personal victimisation (Workman, 2008; Vishwanath et al., 2011, 2016). How does fraud information make the HSM produce invalid cognition and thus affect people’s vulnerability to fraud? Scholars believe that information processing activities are divided into two discrete subprocesses, attention and elaboration (regarded as indicators of systematic processing, Frauenstein and Flowerday, 2020; Gao, 2021). Different degrees of attention to and elaboration of information ultimately lead to different susceptibilities to fraud victimisation.

Attention is the first stage of information processing. This initial attention may cause specific individuals to feel compelled to search for further clues in the email, relate these clues to existing knowledge, determine whether the email is relevant and ultimately conclude that the email is a hoax (Jakobsson, 2007). The research shows that there is a significant correlation between the degree of attention and elaboration. Individuals who pay more attention to information elements have a higher degree of elaboration (Harrison et al., 2016b). For example, suspicious concerns about typographical errors, grammatical errors, and website addresses in phishing emails may lead to more detailed message elaboration, resulting in systematic processing and reducing the likelihood of being victimised by phishing (Toma and Hancock, 2012). Of course, attention to clues focuses more on quality than quantity. Grazioli (2004) found that successful detection does not heed deception cues more than unsuccessful detection, which is different from conventional perception.

In the second stage of the information processing-elaboration process, elaborate information processing occurs when individuals relate these information elements to prior knowledge and experience by adopting a central (systematic) processing path. In contrast, when the peripheral (heuristic) processing path is adopted, no attention is given to the information elements or no elaboration processing is conducted for the noticed information elements (Perse, 1990; Eveland et al., 2003; Gao, 2021). People who elaborate on clues are more likely to understand, learn, retain, and subsequently recall information than those who only focus on clues (Cialdini, 2001; Eveland et al., 2003). Vishwanath et al. (2011) and Harrison et al. (2016b) found that elaboration is a predictor of individual phishing, which is related to a lower likelihood of being victimised by phishing. The elaboration and processing of information content (i.e., using systematic processing) reduce the likelihood of being cheated.

### Factors related to the selection of the heuristic-analytic processing mode

**Figure 2:**
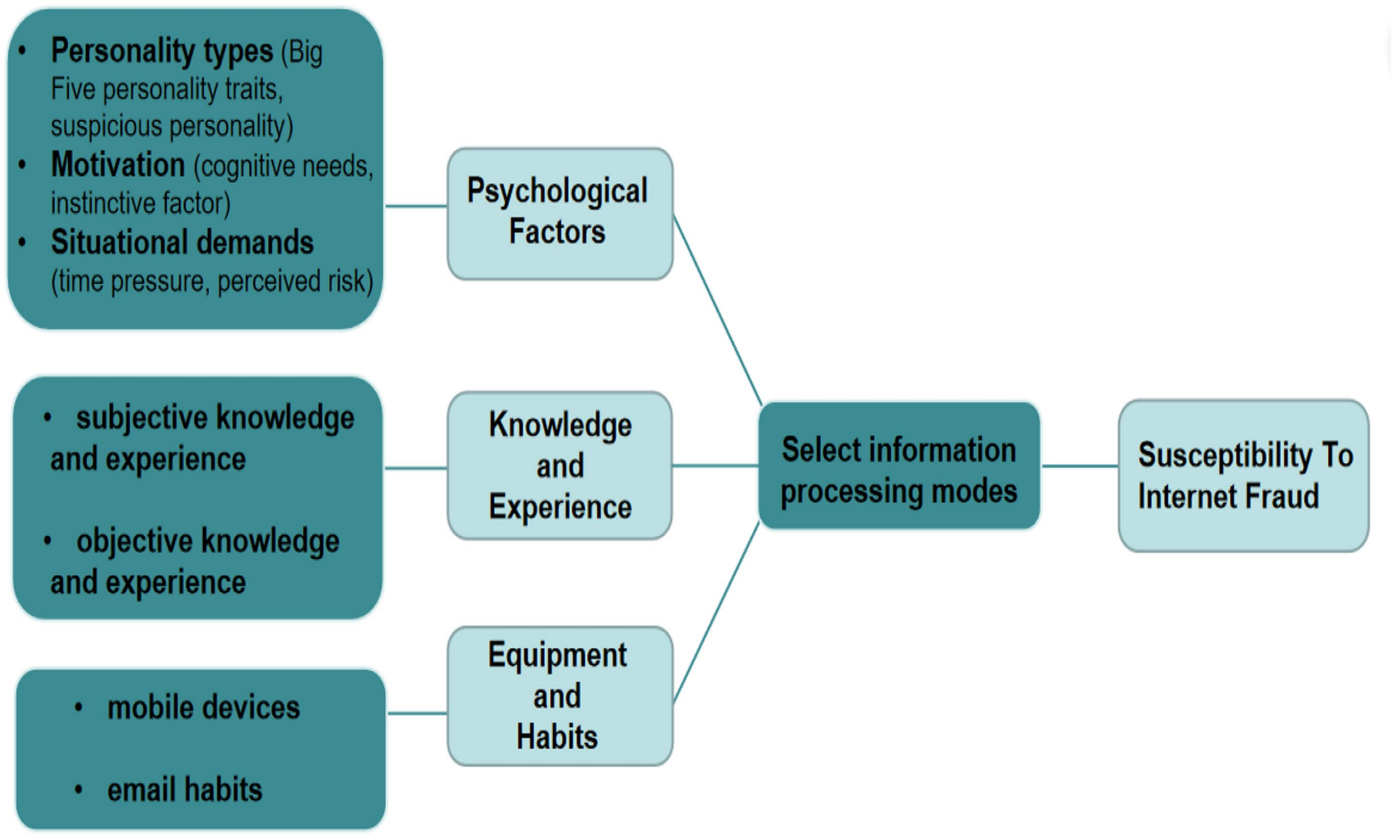
Influencing factors of susceptibility to internet fraud under a framework of the HSM (model drawing came from the author’s construction, and adapted from Wright and Marett, 2010; Norris et al., 2019).

As mentioned in the introduction, research on the influencing factors of online fraud includes demographics, psychological traits and other variables. The discussion in this section explains what factors may influence an individual’s information processing mode and lead to network fraud under the framework of the HSM. Our inductive findings show that psychological factors, knowledge and experience, equipment and habits may influence the cognitive processing mode for internet fraud (the initiation of the heuristic system mode or analytic system mode; Figure 2).

#### Psychological factors

##### Personality type

Research on personality types mainly focuses on the Big Five personality traits and suspicious personality. Studies have been conducted on the relationship between the Big Five personality traits and the likelihood of online fraud victimisation (Norris et al., 2019). For example, Alseadoon et al. (2012) found that individuals with a high degree of agreeableness, openness and extraversion are highly susceptible to information on the internet, but this study did not reach a consistent conclusion. Cho et al. (2016) found that agreeableness and neuroticism had significant predictive effects on the likelihood of being cheated on the internet. Within the framework of the HSM, only the study by Frauenstein and Flowerday (2020) was found. These authors showed that heuristic processing increased the susceptibility to phishing and examined for the first time the effect of the relationship between the Big Five personality model and the heuristic-systematic model of information processing. They found that extraversion was not statistically correlated with either heuristic or systematic processing; agreeableness, neuroticism and openness all had effects with both heuristic and systematic processing; and conscientiousness was statistically correlated with heuristic processing but had no effect with systematic processing. It should be noted that some personality variables (such as agreeableness and neuroticism) are in the same direction as the effects of heuristic processing and systemic processing, which confirms, to some extent, that heuristic processing and systemic processing may be enabled simultaneously when processing information.

There is a tendency among individuals to be suspicious of the intentions of others, which is a type of persistent personality trait and is defined as generalised communicative suspicion (GCS; Levine and McCornack, 1991). Research on the relationship among GCS, the HSM and the susceptibility to network fraud has gone through two stages: in stage 1, GCS and the HSM were regarded as independent dependent variables; in stage 2, the linkage between GCS and the HSM was established. Stage 2 is mainly discussed here. According to the viewpoint of the HSM and internet fraud victimisation, the main reason network users fail to identify fraud and ultimately are victimised is that they start the heuristic system when processing information (Grazioli and Wang, 2001; Wang et al., 2012; Luo et al., 2013; Huang et al., 2022). Harrison et al. (2016a) introduced information insufficiency as a mediator between GCS and the HSM and found that high GCS increases uncertainty and leads to a desire for more information before making a judgement. The desire for more information leads to systematic processing of available information and more accurate detection of phishing deception.

##### Motivation

According to the HSM, if people lack motivation, they tend to limit their investments of time and cognitive resources (Luo et al., 2013). Individuals with the motivation to process information pay attention to key information of arguments and then conduct elaborative processing. In contrast, individuals who lack motivation may focus on cues peripheral to the main argument and may be persuaded by noncontent cues (Petty et al., 1981; Petty and Cacioppo, 1986; Stamm and Dube, 1994). The motivation of receivers to pay attention to information determines the degree of information elaboration. The more motivated network users are to consider a scam, the more likely they are to carefully evaluate the details of the information, which may lead to the discovery of leaked clues about the scam and thus to the avoidance of victimisation (Langenderfer and Shimp, 2001; Wang et al., 2012). Langenderfer and Shimp (2001) also found a potentially negative correlation between motivation and vulnerability to fraud victimisation and suggested that a low level of motivation may be one of the reasons for a lack of review. However, some scholars believe that when individuals are in a state of strong motivation, they do not fully elaborate on the advantages and disadvantages of decision-making, neglect possible problems, and reduce the quality of their decision-making and related information processing (Schwarz et al., 1980; Frey, 1986; Fischer et al., 2008). Experiments by Ariely et al. (2009) confirmed that decision-making deteriorates when the amount involved is large enough to exceed people’s normal experience.

These inconsistent findings require a search for cognitive and instinctive factors in motivation. According to the HSM, the motivation to commit cognitive resources is premised on personal expectations about behaviour (cognitive needs; Chaiken, 1987). Perceived information insufficiency significantly predicts system processing, and the greater cognitive needs are, the greater the need to use processing resources (Vishwanath, 2015). People with higher cognitive needs are less affected by heuristic processing, so they are less likely to be cheated (Luo et al., 2013). However, in some cases, even if motivation is high, people may still be subject to fraud. This may be related to instinct, which often produces thoughtless decisions; that is, people affected by instinct usually do not consider the consequences of their own actions (Loewenstein, 1996). When individuals are too eager to obtain a reward promised by fraudulent information or to avoid the danger contained in fraudulent information, they ignore obvious clues to fraud in the attention process (Langenderfer and Shimp, 2001). Therefore, the influence of motivation on the vulnerability to fraud may be moderated by instinctual factors. When instinct has a great influence, individuals with strong motivation are more likely to miss clues in the information and focus more on rewards or avoiding losses, whereas when instinct has little influence, individuals may choose to carefully evaluate the details of the information rather than the reward itself (Whitty, 2013; Jones et al., 2015).

##### Situational demands (time pressure, perceived risk, etc.)

Shah et al. (2004) found that in phishing emails, people focus disproportionately on urgent cues and tend to ignore other elements, such as the source, grammar, and spelling (Jakobsson, 2007). Attention to urgent cues may induce a sense of urgency and pressure, and individuals under time pressure tend to rely more on one of these cues or use fewer product attributes to make choices, eliminating the systematic processing that requires time and cognitive resources (Wright, 1974; Rothstein, 1986). Information produced by phishers that contains urgent cues reduces the cognitive processing of information and inhibits the systematic processing of other cues that may indicate illegitimate information sources. Phishers hope that these urgent cues will emphasise emotional responses and guide users away from more rational decision-making processes (Workman, 2008; Vishwanath et al., 2011; Harrison et al., 2016b). Luo et al. (2013) proposed that imposing more time pressure on phishing messages may reduce the impact of argument quality and increase the effect of source credibility and the herd effect, thus priming heuristic processing and influencing susceptibility to fraud victimisation. However, some studies have shown that email characteristics (i.e., the need for timely decision-making) do not influence how web users process phishing emails (Harrison et al., 2016b).

Risk-related beliefs have been found to be the most commonly used cognition when individuals examine risk-related actions (Griffin et al., 2002). When people perceive a threat, they adjust their behaviours based on the risk and possible damage caused by the threat (Grothmann and Reusswig, 2006). Individuals anticipate that their behaviours will have serious consequences, which increases their uncertainty, and systematic processing occurs (Workman, 2008). Vishwanath et al. (2016) found that cyber-risk beliefs are negatively related to heuristic processing and positively related to systematic processing. Individuals with strong cyber-risk beliefs are more able to identify online fraud. This is different from the findings of Das et al. (2003), who suggested that the existence of threat elements in information may have a special impact on information processing; as a result, information processing resources are distributed unevenly, and the acceptability of persuasive information increases. Studies have shown that the perceived risk caused by fear does not influence the elaboration process, and some scholars have also verified that higher perceived risk did not decrease the likelihood that a person would be deceived by a phishing email through experiments. This is because when it comes to online fraud, some people with higher perceived risk may fear the consequences of a wrong judgment, and they may be less motivated to detect deception cues because of possible interpersonal and economic repercussions (Wright and Marett, 2010; Harrison et al., 2016b).

#### Knowledge and experience

The stage of information elaboration processing can be predicted by knowledge and experience variables. People who do not have the experience or knowledge necessary to understand an argument usually rely on peripheral clues in the information, which triggers heuristic processing and may lead to incorrect decisions (Petty and Cacioppo, 1986; Chen and Chaiken, 1999). Wright and Marett (2010) found that in the context of phishing, security knowledge and network experience can help users more easily find and identify fraudulent clues in phishing emails, increase the possibility of attention to and elaboration of the information, and thus reduce the possibility of victimisation from phishing. Harrison et al. (2016b) also found that elaboration is not influenced by message factors but is predicted by knowledge in specific fields.

With the growth of acquired knowledge and cognitive skills, people are able to critically analyse relevant information, which makes adults less reliant on heuristic processing than children (Ross, 1981; Petty and Cacioppo, 1986). Knowledgeable subjects are able to participate in and successfully complete deception detection even under time pressure (Grazioli, 2004). Knowledge of email scams increases attention to phishing scam indicators and directly reduces the likelihood of responses (Wang et al., 2012). A higher level of prior professional knowledge among information receivers increases their ability to understand and process relevant issues, which increases the likelihood of elaboration and reduces reliance on peripheral cues (Ratneshwar and Chaiken, 1991).

Harrison et al. (2016b) distinguished between subjective and objective knowledge and found that only objective phishing knowledge was associated with more attention to emails. More knowledge also means that less attention resources are used to trigger professional knowledge. However, it has also been argued that since stored knowledge is often biased towards the original viewpoint, such prior knowledge may provide a biased view of information provided externally (Crocker et al., 1984). False knowledge (subjective knowledge) may also cause a false sense of confidence and lead to decreased attention and elaboration of the specific nuances in phishing emails that may reveal deception (Harrison et al., 2016b).

#### Equipment and habits

Recent research has shown that the use of mobile devices such as smartphones can make people more likely to fall into online fraud traps by enhancing heuristic processing. If users prefer to process emails on their mobile phones rather than computers, they will be more responsive to the heuristic clues contained in phishing emails (Kim and Sundar, 2015; Vishwanath, 2016). Compared with computers, smartphones have smaller screens and are mostly touch based, so content must be displayed in a limited space (Sundar, 2008). The design and layout of smartphones emphasise rich graphical clues rather than text content. Rich presentation exhausts the limited cognitive capacity and resources needed to process persuasive content, thus enhancing heuristic processing (Kim and Sundar, 2015; Vishwanath, 2015). Moreover, a multitasking processing mode reduces the available cognitive resources for system processing (Chaiken, 1987; Ratneshwar and Chaiken, 1991). Experimental results show that a large screen size and video mode of smartphones promotes heuristic processing, while a small screen size and text mode promotes systematic processing (Kim and Sundar, 2015). However, some studies suggest that email habits and cognitive heuristics jointly and independently affect the possibility of being cheated on the internet. Mobile devices such as smartphones affect vulnerability to fraud by strengthening habits rather than affecting cognitive processing (Sundar, 2008; Vishwanath, 2016).

A habit is an automatic response or behaviour pattern that follows a fixed cognitive pattern; it is triggered by environmental stimuli and executed without positive consideration (Bargh and Gollwitzer, 1994; LaRose and Eastin, 2004). Studies have reported that responses to phishing emails can be constricted by habitual response patterns (e.g., responding immediately upon waking up in the morning); that is, individuals respond automatically to relevant emails rather than actively paying attention to them (Vishwanath et al., 2011). Based on the definition of habit, habitual email behaviour that is formulated unconsciously is separate from conscious behaviour that involves some degree of thinking (Aarts et al., 1998). In other words, the habit of replying to online fraud information involves a lack of attention and elaboration of the HSM. Within the framework of the HSM, there are three main ways for email habits to influence online fraud victims: habitual patterns of media usage (an extreme value of involvement, which is positively related to the level of elaboration) combined with a high-level email load (which is negatively related to the level of elaboration) have a strong and significant impact on the likelihood of individuals being phished (Vishwanath et al., 2011); email habits are negatively related to suspicion, heuristic processing is also negatively related to suspicion, and systematic processing is positively related to suspicion (Vishwanath et al., 2011); and email habits are parallel to the heuristic-systematic model (Vishwanath et al., 2016).

### Measures of the heuristic-analytic processing mode, influencing factors and likelihood of internet fraud victimisation

Measures of the likelihood of internet fraud victimisation under the HSM framework are conducted by the experimental method. During these experiments, experimenters provide victims with fraud materials (the materials may be real fraud materials or may be designed by the researchers according to the research purpose), such as shopping websites (Grazioli, 2004), phishing emails (Vishwanath et al., 2011; Luo et al., 2013; Harrison et al., 2016b), and financial statements (Grazioli and Wang, 2001). The subject’s judgement of the validity of these materials, or whether the subject responds, is used as an assessment of the likelihood of being cheated. For example, Wang et al. (2012) investigated 321 members of a public university community in the northeastern United States with a real phishing email as a stimulus. The researchers claimed that they were an email team, notified users of a website upgrade, asked users to verify their email account information, and required users to provide their user name, password and other information. Users were told that if they did not provide the requested information within 7 days, they would permanently lose their email accounts. The title of the email read “UPGRADE YOUR EMAIL ACCOUNT NOW.” In the study by Wang et al., subjects had the possibility of being cheated if they responded to emails and provided information but not otherwise.

Measures of heuristic processing and systematic processing are mainly carried out through self-reports after experiments. Different researchers have designed different contents and quantities of items; some studies used 3 items (Vishwanath et al., 2011), some studies used 4 items (Griffin et al., 2002), and some studies used 6 items (Schemer et al., 2008). The scale of Vishwanath et al. (2011) has often been cited: heuristic processing includes 4 items, such as “I skimmed (i.e., moved quickly) through the Facebook message” and “I briefly looked at the sender/source of the message”; systematic processing includes 3 items, such as “I thought about the action I took based on what I saw in the Facebook message.” Although different scales had different contents and quantities of items, they all adopted a five-point Likert scale.

Attention to and elaboration of the subsystems of the HSM have also been measured by self-reports, but the specific measurement methods were different. Some studies have referred to the scale of Eveland et al. (2003) or Eveland and Dunwoody (2002). Some studies have used alternative methods for measures. For example, Harrison et al. (2016b) used response length (word count) as a measure to capture the level of elaboration, while the degree of elaboration was measured by an open-ended item asking participants why they did or did not do something. For attention, the researchers measured attention to email elements by accurately recalling email elements. They found that elaboration and attention were significantly correlated with each other such that individuals who showed more elaboration of the message also showed more attention to the message elements.

Influencing factors can be measured through existing scales, such as the BFI personality trait scale (John and Srivastava, 1999), suspicion scale (Lyons et al., 2011), suspicion of humanity scale (McKnight et al., 2003; Wright and Marett, 2010), cyber-risk beliefs scale (Vishwanath et al., 2016), risk beliefs scale (Jarvenpaa et al., 2000; Malhotra et al., 2004; Wright and Marett, 2010), perceived risk scale (Drolet and Morrison, 2001; Grazioli and Wang, 2001), domain-specific knowledge scale (Vishwanath et al., 2011), subjective e-mail knowledge and experience scale (Harrison et al., 2016b), web experience scale (Everard and Galletta, 2005; Wright and Marett, 2010), and email habits scale (Verplanken and Orbell, 2003), or influencing factors can be controlled through experiments. For example, Wang et al. (2012) gave a time and fear atmosphere (for example, if the requested information was not provided within 7 days, the users would lose their email accounts indefinitely).

### Defence strategies under the HSM

Within the theoretical framework of the heuristic-systematic model, countermeasures to susceptibility to online fraud mainly include technology, education and simulated scene training.

#### Technology

Huang et al. (2022) suggested that online fraud may involve inherent human weaknesses, such as lack of attention. Based on eye-tracking data, they developed a human-technical solution that generates adaptive visual aids (ADVERT) to direct users’ attention to the email content instead of peripheral cues. They reported success in a case study based on a human experimental dataset from New York University. Chen and Yang (2022) also developed an advanced deep attention collaborative filter to help users analyse social information directly or indirectly to detect spam, which was tested successfully in a case study based on the context of an educational organisation. In addition, previous studies have found that device affordance may affect heuristic processing by leading users to relax their cognitive participation in information processing, reducing their cognitive resource investment, enabling them to perform heuristic processing on cognitive information, and thus making them vulnerable to online fraud (Kim and Sundar, 2015; Vishwanath, 2016). The use of technology to defend against fraud attacks mediated by intelligent devices has also shown positive results, such as spam blockers (Vishwanath et al., 2011), fraud risk identification systems (Frauenstein and Flowerday, 2020), and anti-phishing software and toolbars (Wright and Marett, 2010).

#### Education

Groups with high vulnerability to online fraud are generally characterised by a lack of relevant network security knowledge and poor risk perception. Education can enrich individuals’ network security knowledge reserves and enhance their risk beliefs. The results of current studies show that education is the most promising way to prevent phishing (Wright and Marett, 2010). To implement educational measures, network security knowledge education should be strengthened, such as targeting training and education on email deception detection (Harrison et al., 2016a), legal initiatives to combat internet deception (Grazioli, 2004), and user training efforts (Luo et al., 2013). People who do not have specific domain knowledge are less able to detect deceptive information; they tend to perform peripheral processing and rely on simple clues embedded in emails during information processing and thus make incorrect decisions and suffer from online fraud (Vishwanath et al., 2011). Improved knowledge through education can help people identify fraud clues more easily, increase attention to and elaboration of the information, and reduce the likelihood of being victimised by phishing (Harrison et al., 2016b). Additionally, people’s risk perception ability should be improved through education, such as cyber-risk belief education (Vishwanath et al., 2016), security awareness education programmes (Frauenstein and Flowerday, 2020), and scam awareness training (Wang et al., 2012).

#### Simulated scene training

Simulated scene training is an embedded education method that involves users role-playing on a mocked-up email inbox and being presented with several different scenarios. Participants are exposed to several types of email phishing and are able to experience the results of appropriate and inappropriate responses (Kumaraguru et al., 2007; Sheng et al., 2007; Wright and Marett, 2010). This measure has been officially recognised; for example, to prevent phishing, institutions such as the New York State government have adopted contextual training in which users are sent simulated phishing emails and are given materials on combating phishing at the end of the research (Wright and Marett, 2010). Through lifelike interaction, network users immersed in the simulated network fraud environment can learn relevant anti-fraud knowledge and experience it actively, intuitively and vividly while effectively improving their sense of network self-efficacy. This makes them more confident when processing information related to network fraud in reality and ultimately reduces the likelihood of responding to network fraud information.

## Discussion

### The heuristic system and internet fraud victimisation

The analysed literature seems to agree that network fraud is related to heuristic processing and the analytic processing mode is used to identify fraud. This is because the heuristic system relies on intuition, the parallel processing speed is fast, and decision errors occur easily, whereas the analytic system relies more on rationality, the processing speed is slow, and the error probability is relatively low (Chaiken, 1980; Evans, 2003). This conclusion has also been confirmed by interpersonal deception theory and the theory of deception (Johnson et al., 1992; Buller and Burgoon, 1996; Johnson et al., 2001). However, despite the experimental results of the heuristic-analytic system and vulnerability to online deception, the supporting evidence is not solid.

First, there are few direct empirical studies in the literature (only the three studies reported here: Vishwanath et al., 2016, Harrison et al., 2016b, and Frauenstein and Flowerday, 2020). Second, these three studies do not absolutely support the explanation of online fraud victimisation by the HSM. For example, Frauenstein and Flowerday (2020) did not find that systematic processing has a significant correlation with phishing susceptibility. Third, some researchers do not agree with the division of the two systems in the decision-making and reasoning process. For example, Moshman (2000) suggested that the heuristic system has an implicit nature while the analytic system has an automated nature, and the division of the two systems cannot cover the whole process of decision-making and reasoning. If there is no dual system division, the prediction of the likelihood of network fraud victimisation by heuristic processing is difficult to support. Last but not least, the view that the rational analytic system must be superior to the intuitive heuristic system may be incorrect. On the basis of the assumptions of bounded rationality and ecological rationality, Gigerenzer and the ABC Research Group under his guidance discovered and proposed the “Fast and frugal heuristics” (Gigerenzer, 1996, 2008a,b; Goldstein and Gigerenzer, 2002; Gigerenzer et al., 2008; Liu, 2009). A large number of studies showed that “Fast and frugal heuristics” was reasonable and efficient cognitive strategies to save information. For example, Gigerenzer and Gaissmaier (2011) found that ignoring part of the information could lead to more accurate judgments than weighting and adding all information, for instance for low predictability and small samples. The existence of these uncertain or controversial viewpoints require more effective research to demonstrate the correlation between heuristic information processing mode and network fraud.

According to the views of the scholars in our study, attention and elaboration are regarded as subsystems of the HSM (Frauenstein and Flowerday, 2020; Gao, 2021), and scholars regard attention and elaboration as subsystems of the ELM (Petty and Cacioppo, 1986). Empirical studies also confirm the influence of attention and elaboration on the susceptibility to online fraud (Vishwanath et al., 2011; Toma and Hancock, 2012). It is important to note that attention is not focused on the number of clues but on the quality of the clues, which is used to judge online fraud. Theoretically, the explanation for the HSM is the use of cognitive busyness or cognitive laziness (Petty and Wegener, 1999), adjustment insufficiency (Epley and Gilovich, 2004), and intuitive confidence (Simmons and Nelson, 2006). However, these mechanisms have not been suggested in current studies that adopt the HSM to explain susceptibility to online fraud. In addition, Gigerenzer (2008a) has summarized 10 kinds of “Fast and frugal heuristics “, such as recognition heuristics (if one of the two or more options is recognized, it is inferred that it has a higher validity value), adoption of the best heuristics (search the clue according to the validity of the clue, and terminate the search once the clue that can distinguish the two options is encountered). More evidence is needed to confirm which “quick thrift heuristic” is associated with online fraud victims.

The above discussion does not aim to deny the relationship between the heuristic-analytic system and online fraud victimisation. Despite research on the relationship between the two processing systems and susceptibility to online fraud or on the relationship between the explanation mechanism (the subsystems) of the two processing systems and susceptibility to online fraud, further demonstration is needed.

### Factors related to the selection of the heuristic-analytic processing mode

In our research, exploration of the influencing factors was conducted within the HSM framework, which is different from simply studying the influencing factors of susceptibility to online fraud. In the process of analysis, psychological factors are unstable variables, and different studies have mutually exclusive results. For example, in the study of motivation, generally speaking, individuals with the motivation to process information pay attention to key information arguments and then carry out elaboration processing (Luo et al., 2013). However, decisions deteriorate when the amount of information involved is large enough to exceed individuals’ normal experience (Ariely et al., 2009). Risk perception under situational demand involves uncertainty, which may be caused by different definitions of risk perception. If risk perception is regarded as a permanent personality, individuals with strong cyber-risk beliefs may be able to activate systematic processing to better identify online fraud (Vishwanath et al., 2016). However, if there is a state of fear caused by threat elements in the information, it may increase vulnerability to deception (Das et al., 2003) or have no influence (Das et al., 2003; Wright and Marett, 2010).

In addition, since personality traits have been applied to the HSM framework for the first time (Frauenstein and Flowerday, 2020), their mechanism needs to be further explored. In contrast to the above factors, high GCS increases uncertainty, which leads to the systematic processing of available information and more accurate phishing detection (Harrison et al., 2016a). This finding is consistent with previous studies (Wright and Marett, 2010). Are there other psychological factors that influence the selection of the heuristics and analytic systems?

Forgas and East (2008) found that the emotions of online users affect their ability to detect deception. When users feel sad, their detection ability improves. According to the ELM, under relatively low thinking conditions, similar to other variables, emotions can affect attitudes through various low effort processes. However, when the likelihood of thinking is relatively high, these same emotions can affect persuasion through other mechanisms (Petty and Briñol, 2014). Whether emotions affect susceptibility to online fraud by influencing the mediating effect of the heuristic and systematic processing modes needs to be further explored. Building workers often live far from their families, which can lead to loneliness over time (Schonfeld and Chang, 2017). A survey found that to eliminate loneliness and insecurity, they chose to make friends online, which led to online cheating in relationships.

Within the framework of the HSM, a relatively consistent conclusion is that knowledge and experience, especially the specific knowledge and experience related to online fraud, are protective factors against online fraud (Wright and Marett, 2010). Interpersonal deception research puts experience at the centre of the fraud detection process; experience can improve the accuracy of identifying deceptive information (Feeley et al., 1995). When relevant events are stored and easily accessible, it is easier to make connections between the information received and relevant events, so those with relevant knowledge and experience are better able to process new information elaborately. Two points should be noted: (a) knowledge can be divided into subjective knowledge and objective knowledge, with more emphasis on objective knowledge (Harrison et al., 2016b), and (b) prior knowledge may involve a biased review of externally provided information (Crocker et al., 1984).

The influence of device affordance and habits on online fraud victimisation is a relatively new area of research. Previous studies have found that a large screen size and video mode of smartphones facilitate heuristic processing, while a small screen size and text mode facilitate systematic processing (Kim and Sundar, 2015). However, Vishwanath (2016) suggested that mobile devices such as smartphones have an impact on the susceptibility of fraud victims by reinforcing habits rather than affecting cognitive processing. This requires consideration of a deeper question of whether habits affect information processing patterns. Regarding online fraud, this research is lacking and needs to be further enhanced.

### Measures of the heuristic-analytic processing mode, influencing factors and the likelihood of internet fraud victimisation

The validity and reliability of the scales used were not reported, although there are scales to measure the heuristic and systematic processing modes (including attention and elaboration). Scales to measure the influencing factors were previously available and are not discussed here. We mainly discuss the data collection method used in the research, the simulation experiment. First, this method of data collection is generally agreed upon by experimental subjects in advance, so there are no ethical issues. However, the participants’ environment, the expectation of the stimulating nature of the experiment, the degree of attention, the loss when making incorrect decisions and other factors are very different from real online fraud (Jones and Towse, 2018; Gao, 2021). Second, whether users click the link in phishing emails (Luo et al., 2013; Harrison et al., 2016b) and whether they provide the private information requested in phishing emails (Wang et al., 2012) are used to measure vulnerability to online fraud, which is not equivalent to ultimately being cheated. Third, the subjects used in the experiments were ordinary people (Vishwanath et al., 2011) rather than real victims. Although there may be self-report bias when real victims are used as subjects, this situation is more realistic and objective in terms of influencing factors.

### Defence strategies under the HSM

Compared with the defence strategies proposed in the literature that are included in our analysis, previous defence strategies in the non-HSM framework focused on two aspects: technology and education. However, in the framework of the HSM, defence technology for online fraud is more prominent in guiding potential victims to initiate the systematic processing mode (traditional technology emphasises internet fraud information blocking from the government, internet providers, shopping and other related websites). For example, Huang et al. (2022) developed a human-technical solution that generates adaptive visual aids (ADVERT) to direct the user’s attention to the email content instead of peripheral cues. In education, while attaching importance to knowledge and experience, some researchers have proposed simulated scene training (Kumaraguru et al., 2007; Sheng et al., 2007). Through lifelike interaction, network users immersed in a simulated network fraud environment can learn relevant anti-fraud knowledge and experience it more actively, intuitively and vividly, effectively improving their sense of network risk perception and self-efficacy and avoiding the cognitive load caused by intensive publicity and education (Williams and Noyes, 2007).

However, simulated scene training also suffers from certain challenges. For example, when simulated phishing studies are used, participants who choose to respond to emails may feel embarrassed and upset because they demonstrate the same vulnerability as real-life victims (Jones et al., 2015). Some scholars have suggested that participants participate in the simulation scenario in an informed manner and conduct the internet fraud attack test after a period of time. However, these problems may still occur if the participants are subjected to an online fraud attack test after they forget they have joined, and the possibility of users responding will be reduced if the participants are fully informed (Mack, 2014). Therefore, in the future, it is necessary to further optimise the simulation scenario and to improve the simulation process and the simulation education effect.

## Limitations of this review

The current systematic review is not without limitations. On the one hand, due to keyword selection and database limitations, the number of studies that met the selection criteria was small. Therefore, this study may not cover all the research on online fraud under the HSM framework. The current research only includes articles published in peer-reviewed journals and written in English. Future research could incorporate papers published in other venues (e.g., conference papers) or could further systematically review papers published in other languages on this subject. Nevertheless, this study reviews the relationship between individual information processing modes and online fraud victimisation, influencing factors, heuristic and analytic systems and their explanatory mechanisms, measures of influencing factors, and defence strategy, laying a theoretical foundation for research in this field. In addition, some research gaps were found in this study that provide a direction for future work in this new research area. This study did not conduct a statistical significance level test and effect size determination on the results of previous studies and involved a systematic review rather than a meta-analysis. This is mainly because there were few included articles and different research directions, which could not meet the preconditions for meta-analysis (Cheung and Vijayakumar, 2016). Meta-analytical research is encouraged when a sufficient number of studies share similar research types and variables.

## Conclusion

The two systems of decision-making and reasoning are in the initial stages of explaining online fraud victimisation; nevertheless, they show that online fraud victimisation may be related to humans’ inherent weakness in decision-making. When individuals face online fraud information, if they activate the heuristic processing mode to process the information, they may increase the likelihood of victimisation. According to the defence strategy under the HSM, technical application that emphasises directing users’ attention to the content of emails as well as immersive simulated scene training may provide a major breakthrough in combating online fraud in the future. However, the verification of the heuristic and analytic processing modes for the prediction of network fraud victimisation as well as the explanatory mechanism and influencing factors need to be further expanded.

## Author contributions

YS: ideas, data collection, writing, and revisions. KW: writing and revisions. YT and YZ: revisions and analysis. BM: writing and data collection. SL: writing, ideas, and data collection. All authors contributed to the article and approved the submitted version.

## Funding

This work was supported by the Legal Construction and Legal Theory Research Project of China (grant no. 20SFB4038).

## Conflict of interest

The authors declare that the research was conducted in the absence of any commercial or financial relationships that could be construed as a potential conflict of interest.

## Publisher’s note

All claims expressed in this article are solely those of the authors and do not necessarily represent those of their affiliated organizations, or those of the publisher, the editors and the reviewers. Any product that may be evaluated in this article, or claim that may be made by its manufacturer, is not guaranteed or endorsed by the publisher.
