# Supplementary material for: Theoretical basis and occurrence of internet fraud victimisation: Based on two systems in decision-making and reasoning
Source: Front Psychol. 2023 Feb 6;14:1087463. doi: 10.3389/fpsyg.2023.1087463 (PMC9940837; doi:10.3389/fpsyg.2023.1087463)
Supplement: Supplementary file 1 [file Data_Sheet_1.PDF]

**A literature review of theoretical basis and occurrence mechanism of the Internet fraud victimization**

| Authors | Year | Location | Method | Sample | Key findings |
|---|---|---|---|---|---|
| Vishwanath et al. | 2011 | USA | Experimental | Undergraduate students at a large university in the northeast of USA (n=321) | Individuals fall victim to phishing because of a lack of cognitive involvement rather than a lack of ability. Habitual patterns of media use combined with high levels of email load lead people to make decisions based on simple cues and be more likely be deceived. |
| Vishwanath et al. | 2016 | USA | Experimental | Undergraduate students at the University at Buffalo (study1:n=125, study2:n=220) | Phishing susceptibility is affected by suspicion, suspicion is more likely when individuals systematically process the email and less likely when they heuristically process emails. Suspicion influences Phishing Susceptibility. |
| Harrison et al. | 2016a | USA | Experimental | Undergraduate students from a large northeastern public university in USA (n=192) | Suspicious personality leads to a desire for more information, desiring more information leads to systematically processing available information and more accurate phishing deception detection. |
| Harrison et al. | 2016b | USA | Experimental | Undergraduate students at a large university in the northeast of USA (n=194) | Elaboration of the message was greater in individuals who had increased experience and a more global understanding of e-mail. Greater elaboration of the message increased resilience to the phishing attack. |
| Luo et al. | 2013 | USA | Experimental | Faculty and staff members in a public university in the southwest of USA (n=105) | A high level of argument quality that can withstand message recipients' systematic processing increases the likelihood of phishing victimization. The Heuristic Systematic Model provides a theoretical framework for investigating the psychological mechanism of the effectiveness of phishing attacks. |

| | | | | | |
|---|---|---|---|---|---|
| Vishwanath | 2016 | USA | Experimental | undergraduate students enrolled in the University at Buffalo's Singapore program (n=104) | The heuristic processing and habits both simultaneously and independently lead to phishing victimization, but the reliance on heuristics was not greater on mobile devices. Study shows a moderating role of device affordances on email habits rather than heuristic processing. |
| Petty & Briñol | 2014 | USA | Review | | When elaboration is low, emotions can serve as simple clues; When elaboration is high, emotions can be used as an argument; When elaboration is high, emotions can bias cognition; When elaboration is unrestricted, emotions affect thinking. |
| Jones et al. | 2015 | UK | Review | | In the rational decision-making condition, participants correctly identified more email scams than in the intuitive decision-making condition. In high cognitive load situations, people process emails tend to use intuitive, immediate, and emotional responses, while in low cognitive load situations, people process emails tend to use more analytic and deliberate processes. |
| Wright & Marett | 2010 | USA | Experimental | Undergraduate students at a large university in the northwest of USA (n=299) | Higher CSE, increased Web experience, increased security knowledge, and a higher suspicion of humanity all had significant negative effects on deception success. |
| Grazioli & Wang | 2001 | USA | Experimental | Undergraduate students at a large university in USA (n=93) | Internet consumers are able to detect fraud clues, but are unable to effectively combine and evaluate these clues to draw correct conclusions from them. |
| Canfield et al. | 2016 | USA | Experimental | Participants from U.S. Amazon Mechanical Turk (experiment 1: n=152; experiment 2: n=100) | Phishing-related decisions are sensitive to individual detection ability, response bias, confidence and perception of consequences. |

| | | | | | |
|---|---|---|---|---|---|
| | | | | | In the phishing-related decision-making process, the detection task (determining whether an e-mail is legitimate) and the behavioral task (determining how to handle an e-mail message) are interwoven. |
| Johnson et al. | 2001 | USA | Experimental | Auditors (partners in international public accounting firms) (n=24) | Knowledge that supports high base-rate tasks is more likely to be fixed correctly, while knowledge that supports low base-rate tasks is more likely to contain errors. Similarity, simplicity, non-interference and credibility are the characteristics of knowledge that affect fraud detection. |
| Wang et al. | 2012 | USA | Experimental | Undergraduate students at a public university community in the northeast of USA (n=321) | Visceral triggers (such as stressing the urgency to respond) reduce the recipients' depth of information processing and induce recipients to make decision errors (responses to phishing emails), while attention to phishing deception indicators (such as grammar error) decreases the likelihood to make decision errors. Activating and using proper stored heuristics for phishing detection may rely on knowledge of email-based scam. |
| Frauenstein & Flowerday | 2020 | South Africa | Experimental | Final-year undergraduate students at a South African university (n=215) | Heuristic processing increases susceptibility to phishing. Conscientious users have a negative influence on heuristic processing, thus less susceptible to phishing. |
| Chen & Yang | 2022 | China | Modeling And Experimental | a list of objects about a case study applied in the environment of educational organizations (n=25) | Advanced deep attention collaborative filter for secure academic email services can effectively protect against phishing attacks. |
| Huang et al. | 2022 | USA | Data analysis and Experimental | Undergraduate students at New York University (n=160) | Visual aids can increase the attention level and improve the accuracy of phishing recognition. The meta-adaptation can further |

| | | | | | |
|---|---|---|---|---|---|
| | | | | | improve the accuracy of phishing recognition. |
| Valecha et al. | 2022 | USA | Data analysis and Experimental | Phishing emails from an anti-phishing service Millersmile[6](n=17902) legitimate emails from the Enron corpus (n=19153) | Phishers often use persuasion techniques to get positive responses from the recipients. Gain and loss persuasion cues can effectively detect phishing emails. |
